# Machine Learning–Based Evaluation of Combined EBV and CMV Serostatus as Predictors of Post-Transplant Lymphoproliferative Disorder

**DOI:** 10.3389/ti.2026.15781

**Published:** 2026-02-11

**Authors:** Ghazal Azarfar, Muath A. M. Alotaibi, Yingji Sun, Shahid Husain, Aman Sidhu, Mamatha Bhat, Seyed M. Hosseini-Moghaddam

**Affiliations:** 1 Ajmera Transplant Centre, University Health Network, Toronto, ON, Canada; 2 Division of Infectious Diseases, Department of Medicine, University Health Network, University of Toronto, Toronto, ON, Canada; 3 Department of Medicine, King Faisal Specialist Hospital and Research Centre, Madinah, Saudi Arabia; 4 Toronto General Hospital Research Institute (TGHRI), University Health Network, Toronto, ON, Canada; 5 Division of Gastroenterology and Hepatology, Department of Medicine, University of Toronto, Toronto, ON, Canada

**Keywords:** cytomegalovirus (CMV), Epstein–barr virus (EBV), machine learning, post-transplant lymphoproliferative disorder (PTLD), solid organ transplantation

## Abstract

Post-transplant lymphoproliferative disorder (PTLD) is a major complication of solid organ transplantation (SOT), with the greatest risk in Epstein–Barr virus (EBV) donor-positive/recipient-negative (D+/R−) pairs. The contribution of cytomegalovirus (CMV) serostatus is less well defined. We conducted a population-based study of 47,333 abdominal SOT recipients in the United States (1995–2015) using linked SRTR data. Donor–recipient EBV/CMV serostatus was evaluated as a compound variable. The primary outcome was PTLD incidence, with secondary analyses assessing predictors of PTLD and impact on survival. Overall, 716 patients (1.5%) developed PTLD at a median of 6.1 years (IQR 2.9–9.7) after transplant. EBV D+/R- recipients had the highest incidence (3.2%), and those with compound [EBV D+/R−, CMV D−/R−] serostatus had more than double the PTLD risk compared with [EBV D+/R−, CMV D+/R−] (5.3% vs. 2.5%, p < 0.001). Logistic regression and random forest models consistently identified [EBV D+/R−, CMV D-/R-] serostatus, age, and race as leading predictors, though discrimination was modest (test AUC ∼0.61). In a matched survival analysis, PTLD was not associated with increased all-cause mortality (aHR ∼1.0). Our findings demonstrate that combined EBV/CMV serostatus improves PTLD risk prediction compared with EBV alone and emphasize the need for targeted preventive strategies.

## Graphical Abstract

**Figure:**
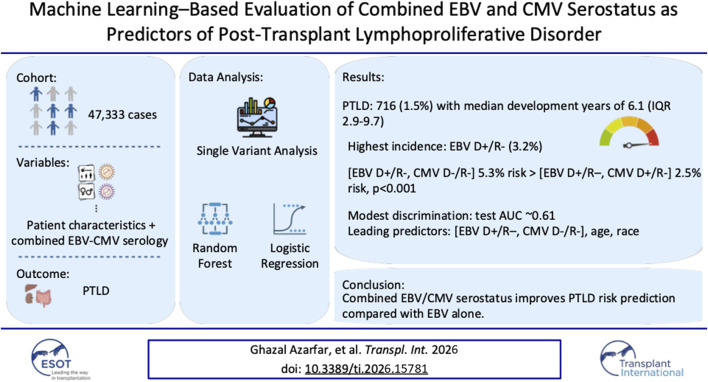

## Introduction

Post-transplant lymphoproliferative disorder (PTLD) is a potentially life-threatening complication that occurs in approximately 1%–16% of solid organ transplant (SOT) recipients [1–4]. The intensity of immunosuppression and Epstein–Barr virus (EBV) replication are key drivers in the development of PTLD [1, 5]. The risk of PTLD is particularly high in EBV-seronegative recipients who acquire primary EBV infection post-transplant, especially when the donor is EBV-seropositive, highlighting the pivotal role of EBV serostatus in PTLD development [6]. The association between cytomegalovirus (CMV) infection and PTLD remains inconclusive, with inconsistent findings across studies. Some studies have identified CMV disease and CMV-EBV coinfection as risk factors for PTLD, while others found no consistent association after adjusting for EBV serostatus and immunosuppression intensity [7–12]. The association between CMV infection and post-transplant outcomes, including PTLD, may be confounded or modified by antiviral prophylaxis [13, 14]. Although some data showed antiviral agents such as valganciclovir may delay EBV viremia, the role of antiviral prophylaxis in preventing PTLD remains debated [15, 16]. A systematic review found insufficient evidence to support this conclusion [17–19]. However, a recent meta-analysis suggests antiviral prophylaxis may reduce PTLD risk and EBV viremia, especially in patients receiving intensive immunosuppression [20].

Inconsistencies in prior observational studies evaluating PTLD risk likely reflect methodological limitations, including small sample sizes, short follow-up durations, single-center designs, and lack of adjustment for CMV serostatus. In this study, we applied machine learning (ML) to estimate PTLD risk in SOT recipients, incorporating EBV-CMV serostatus as a compound variable to explore whether patients with [EBV (D+/R−), CMV (D−/R−)] serostatus are at elevated risk of PTLD.

## Materials and Methods

### Study Design and Population

In this large-scale, population-based study, we included all recipients of abdominal organ transplants (liver, kidney, kidney-pancreas, and intestine) between May 1995 and March 2015, for whom both the recipient’s and the donor’s EBV and CMV serostatus were documented. We used the linked health-related data included in the Scientific Registry of Transplant Recipients (SRTR), which comprises information from every transplant and organ donation that has taken place in the United States since October 1987.^1^ The SRTR provides information on donor-recipient matching and transplant recipients’ demographics, clinical data, and outcomes, as supplied by the Organ Procurement and Transplantation Network (OPTN) and managed by the United Network for Organ Sharing (UNOS). We linked various variables and datasets in the SRTR, including transplant recipient information, registration records, follow-up data, immunosuppressive therapies, and malignancy reports. To minimize bias, we excluded variables with >20% missingness and thoracic transplant recipients, as EBV/CMV serostatus was frequently unreported in this group.

### Exposure and Outcomes

The exposure of interest was the combined EBV and CMV donor/recipient (D/R) serostatus at the time of transplantation, treated as a compound variable. The primary outcome was the development of PTLD, and the secondary outcome was all-cause post-transplant mortality. The observation period extended through 30 November 2023.

### Statistical Analysis

Categorical variables were presented as proportions, and continuous variables as medians with interquartile ranges (IQR). We calculated the cumulative incidence of PTLD across 16 EBV/CMV compound variable categories. Associations between PTLD and categorical variables were assessed using the Chi-square test, while the Mann-Whitney U test was used for continuous variables. A p-value <0.05 was considered statistically significant. Variables significantly associated with PTLD were used as input features for two machine learning algorithms—logistic regression (LR) and random forest classifier (RFC)—to predict PTLD development. Due to the low incidence of PTLD, random undersampling was used to achieve a balanced 1:1 ratio of patients with and without PTLD [21]. The resulting dataset was then randomly split into 80% for training and 20% for testing. This process was repeated 20 times, and the mean and standard deviation of the area under the curve (AUC) and accuracy were calculated for both models on the training and test sets. The results of the LR analysis were reported as adjusted odds ratios (aOR). To interpret model performance and identify the relative importance of predictors, Shapley Additive Explanations (SHAP) analysis was performed for both the LR and RFC models [22, 23]. We estimated the confidence intervals using 200 bootstrap resamples.

Using survival analysis, we determined the all-cause post-transplant mortality risk as the secondary outcome. To account for immortal time bias, we matched each patient diagnosed with PTLD with four patients who did not develop PTLD and survived until the time of PTLD diagnosis in the corresponding case. Matching variables included transplant year (±2.5 years), age (±5 years), sex, race, organ transplanted, and the presence of comorbidities [24]. Matching was achieved by filtering patients based on predetermined matching variables, followed by a random selection of non-PTLD patients for each PTLD case. Patients were censored at the time they were lost to follow-up (i.e., right-censoring). We used the log-rank test and the Kaplan-Meier analysis to compare survival differences between patients with PTLD and patients without PTLD. Cox proportional hazards regression analysis was performed to identify risk factors associated with mortality. All analyses were conducted using Python (version 3.12.4, packaged by Anaconda, Inc.) with the Scikit-learn (sklearn) and scikit-survival (sksurv) libraries.

## Results

During the study period, a total of 47,333 patients underwent abdominal organ transplants (median [IQR] age: 49 [37–58] years, male: 28,718 [60.6%]). Cohort characteristics are provided in Table 1.

**TABLE 1:** Patients’ characteristics in the cohort.

​	Patients without PTLD (n = 46,617)	Patients with PTLD (n = 716)	p Value
Sex, male, *n* (%)	28,262 (60.6)	456 (63.7)	**0.096**
Age (years), median (IQR)	49 (37–58)	52 (42–61)	**<0.001**
BMI, median (IQR)	27 (23–31)	26.5 (23–30)	0.115
Recipient ethnicity (no. [%])			
White	31,918 (68.5)	559 (78.1)	**<0.001**
Black	6,481 (13.9)	54 (7.6)	**<0.001**
Hispanic	5,659 (12.1)	55 (7.7)	**<0.001**
Asian	1,940 (4.2)	26 (3.6)	0.969
Other	619 (1.3)	21 (2.9)	0.026
Previous malignancy (no. [%])	378 (0.8)	6 (0.8)	0.135
Drug treated COPD	537 (1.2)	7 (1.0)	0.670
Drug treated systemic hypertension	34,984 (75.0)	454 (63.4)	0.478
Symptomatic peripheral vascular disease	2,046 (4.4)	33 (4.6)	0.762
Symptomatic cerebrovascular disease	983 (2.1)	21 (2.9)	0.148
Peptic ulcer disease	1,349 (2.9)	23 (3.2)	0.637
Organ transplanted			
Intestine	3 (0.0)	0 (0.0)	-
Kidney	43,215 (92.7)	630 (87.9)	**<0.001**
Liver	2,082 (4.5)	55 (7.7)	**<0.001**
Kidney-pancreas	1,317 (2.8)	31 (4.3)	**0.016**
Post-transplant immunosuppression			​
Cyclosporine, N (%)	5,310 (11.4%)	77 (10.7)	0.263
mTOR inhibitor, N (%)	3,953 (8.5%)	52 (7.3)	0.407
Tacrolimus, N (%)	38,523 (82.6%)	611 (85.3)	**0.035**
Corticosteroid, N (%)	32,171 (69.0%)	480 (67.0)	0.510
Azathioprine, N (%)	631 (1.4%)	20 (2.8)	**<0.001**
Mycophenolate mofetil, N (%)	40,377 (86.6%)	615 (85.9)	0.772
Any anti lymphocyte exposure in the first year, N (%)	898 (1.9%)	9 (1.3)	0.051

Bold values: statistically significant.

### PTLD

A total of 716 patients (1.5%) developed PTLD. The median time to PTLD diagnosis was 6.1 years (IQR 2.9–9.7 years), with 86 patients (12%) developing PTLD within the first year post-transplant. The predominant pathological PTLD subtype was monomorphic PTLD (n = 279 [39.0%]). Supplementary Table 1 provides disease characteristics in SOT recipients with PTLD. Among patients with early-onset PTLD (i.e., diagnosed within 2 years post-transplant), 62 of 142 (42.2%) had extranodal involvement, compared to 112 of 573 (19.5%) among those with late-onset PTLD (P < 0.0001).

The incidence of PTLD in the EBV D+/R− group (137/4,236 [3.23%]) was significantly higher than in the other serogroups (EBV D-/R-: 11/1,073 [1.02%]; EBV D−/R+: 35/2,905 [1.20%]; EBV D+/R+: 533/38,979 [1.36%]; p < 0.001) regardless of the timing of the PTLD development (see Supplementary Tables 4, 5). Table 2 presents the PTLD incidence considering compound variable of [EBV (D/R), CMV (D/R)]. Among EBV mismatch recipients, those with CMV mismatch serostatus (CMV D+/R−) had a significantly lower incidence of PTLD compared to those with CMV D−/R− status [23/938 (2.45%) vs. 67/1,332 (5.3%), p < 0.001]. The lowest PTLD cumulative incidence was in the [EBV (D-/R+), CMV (D+/R-)] recipients (0.57%).

**TABLE 2:** EBV-CMV serology status of the recipients (R) and the donors (D).

​	EBV D−/R−			EBV D+/R−		
	Patients with PTLD (11)	Patients without PTLD (1062)	Cumulative incidence	Patients with PTLD (137)	Patients without PTLD (4239)	Cumulative incidence
CMV D−/R−	5	457	1.08%	67	1265	5.03%
CMV D+/R−	2	145	1.36%	23	915	2.45%
CMV D-/R+	2	280	0.71%	21	758	2.69%
CMV D+/R+	2	180	1.10%	26	1301	1.96%
​	EBV D−/R+			EBV D+/R+		
​	Patients with PTLD (35)	Patients without PTLD (2870)	Cumulative incidence	Patients with PTLD (533)	Patients without PTLD (38446)	Cumulative incidence
CMV D−/R−	15	853	1.73%	140	9089	1.52%
CMV D+/R−	2	350	0.57%	82	6414	1.26%
CMV D−/R+	9	838	1.06%	114	8441	1.33%
CMV D+/R+	9	829	1.07%	197	14502	1.34%

The Chi-square test yielded a global p-value <0.001, demonstrating a significant association between combined EBV–CMV serostatus and PTLD.

In multivariable LR model, we estimated the training accuracy of 61.172 %± 0.010% and the training AUC of 0.658 ± 0.001. The test accuracy and test AUC for the LR were 57.17% ± 0.022% and 0.605 ± 0.023, respectively. Supplementary Table 2 presents the adjusted OR in LR analysis. In the multivariable RFC model, we estimated a training accuracy of 60.658 ± 0.009, with a training AUC of 0.653 ± 0.006. The RFC test accuracy and AUC were 58.104 %± 0.018% and 0.615 ± 0.018, respectively (Table 3).

**TABLE 3:** Comparison of models’ performances. The average of 20 under sampling.

Model	Training set		Test set	
	AUC (95% CI)	Accuracy (95% CI)	AUC (95% CI)	Accuracy (95% CI)
Logistic regression	0.658 (0.657–0.659)	61.172 (61.162–61.182)	0.605 (0.582–0.628)	57.170 (57.148–57.192)
Random forest	0.653 (0.647–0.659)	60.658 (60.649–60.667)	0.615 (0.597–0.633)	58.104 (58.086–58.122)

### SHAP Analysis

**FIGURE 1:**
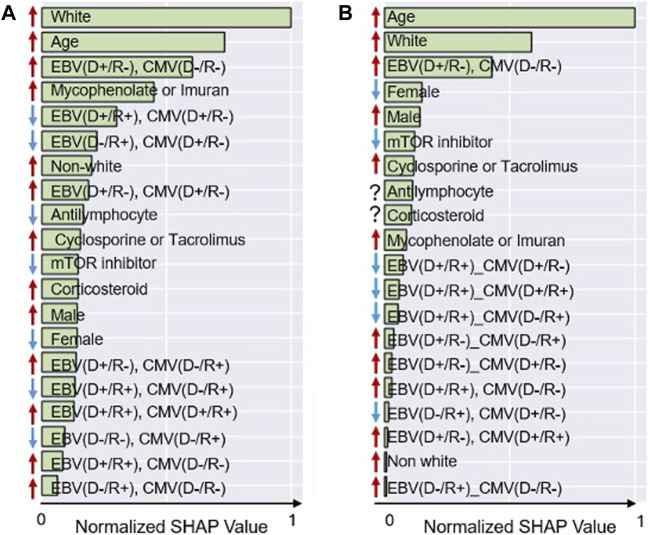
SHAP Analysis, impact of the features on model output. **(A)** Logistic regression. **(B)** Random forest. This figure illustrates the variable importance for the top 20 variables and the association with PTLD. Higher average SHAP values indicate a greater contribution of the variable to the model. The arrows indicate the direction of the association: red arrows represent positive associations, while blue arrows represent negative associations.

**FIGURE 2:**
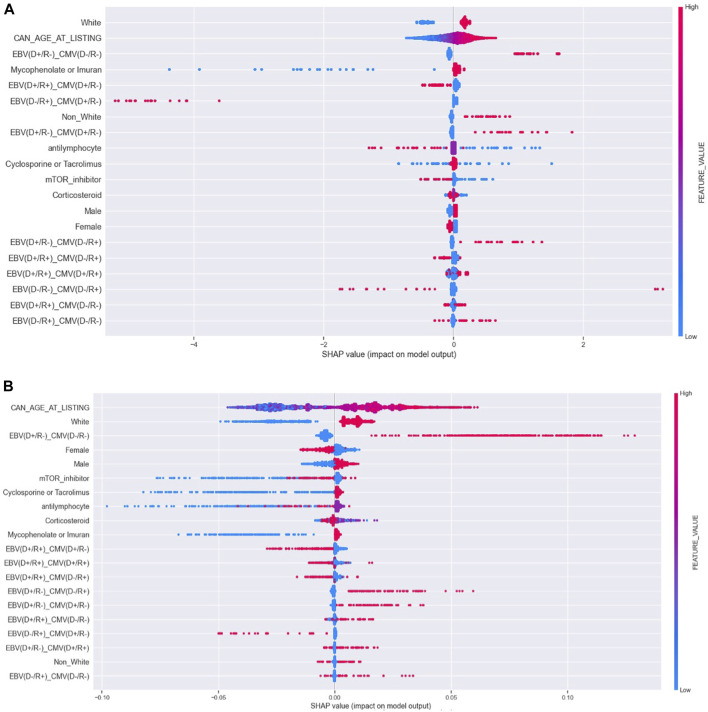
Bees worm Plot of the shapely valued for the LR and RFC analyses. **(A)** Bees worm Plot of the shapely valued for the LR model, **(B)** Bees worm Plot of the shapely valued for the RFC model.

Figure 1 presents the SHAP rankings of the variables used in each model, highlighting the top 20 predictors of PTLD. In both models, age, white race, and combined serostatus—specifically [EBV (D+/R-), CMV (D-/R-)]—consistently emerged as the most influential predictors. The variables incorporated into the two machine learning algorithms are presented in Supplementary Table 3. The rankings of other predictors varied between the LR and RFC models. Male sex and the use of immunosuppressive agents such as mycophenolate compounds, azathioprine, cyclosporine or tacrolimus were associated with PTLD development, while treatment with mTOR inhibitors showed an inverse relationship. Figure 2 provides Bees worm plots of the shapely valued for the LR and RFC analyses.

### All-cause Post-transplant Mortality

In this analysis, 570 patients with PTLD were matched to 2,280 controls without PTLD. Using Cox proportional hazards models, including stratified analyses by age, sex, and race, PTLD was not associated with increased all-cause mortality. Adjusted hazard ratios ranged from 0.991 to 1.008, with 95% confidence intervals consistently crossing unity (e.g., aHR 1.004 [95% CI, 0.900–1.120]; aHR 0.991 [95% CI, 0.883–1.113]). White recipients had a higher risk of mortality compared to non-White recipients (aHR 1.153 [95% CI, 1.027–1.295]). Model discrimination was limited (C-index ∼0.50), indicating no strong mortality signal associated with PTLD in this matched cohort. Kaplan–Meier analysis and log-rank testing showed no significant survival difference between patients with and without PTLD (P = 0.261; Supplementary Figure 1).

## Discussion

EBV-naïve recipients are exposed to donor-derived EBV infection and are vulnerable to PTLD due to unregulated viral replication under immunosuppressive conditions [1]. Previous studies consistently showed patients with EBV D+/R− serostatus are at the greatest risk of PTLD development [25–27]. However, the relationship between CMV serostatus and PTLD has not been thoroughly investigated. Using two ML models, we found a consistent association between [EBV [(D+/R−), CMV (D−/R−)] serostatus and increased PTLD risk. Among EBV mismatch recipients, those with CMV D+/R− had a significantly lower risk of PTLD compared to those with CMV D−/R−. Although single-center studies have suggested a potential link between PTLD and CMV infection, the association between PTLD and CMV serostatus has not been systematically investigated [10, 28, 29]. An early study by Walker et al. in 1995 examined a cohort of 381 non-renal SOT recipients at the Mayo Clinic and proposed a possible role for CMV serostatus in PTLD pathogenesis. However, the interpretation of these results is constrained by confounding from concurrent use of potent immunosuppressive therapy, notably OKT3, which substantially elevates PTLD risk [9]. Opelz et al. conducted a large SRTR-based cohort study (1999–2007) of 23,340 SOT recipients and found no significant association between recipient CMV serostatus and non-Hodgkin lymphoma; however, donor CMV serostatus was not included in their analyses [30]. In a separate cohort study, Geris et al. found a relationship between CMV D-/R- serostatus and the development of PTLD. However, the study outcome was limited to diffuse large B-cell lymphoma (DLBCL) and did not investigate the simultaneous roles of EBV (D/R) and CMV (D/R) as a compound variable [7]. Ali et al., in a cohort of 10,947 pediatric renal transplant recipients, found that recipient CMV seropositivity was associated with a protective effect against PTLD (HR = 0.82; 95% CI: 0.73–0.94) [11]. To our knowledge, no prior study has evaluated the association between PTLD and combined EBV/CMV donor–recipient serostatus as a single compound variable.

We evaluated PTLD risk using combined [EBV (D/R), CMV (D/R)] serostatus and found that EBV D+/R- recipients with CMV D−/R− serostatus were at increased risk of PTLD. This observation does not inherently indicate a protective effect of valganciclovir, which is typically not administered to CMV D-/R- recipients. Although they may receive acyclovir or valacyclovir, these agents have negligible activities against CMV and no proven benefit in preventing EBV-related PTLD. Aldabbagh et al. conducted a metanalysis in 2017 including 9 studies and 2,366 SOT recipients and concluded that existing data are insufficient to support the routine use of antivirals to prevent PTLD in EBV-naive SOT recipients [19]. However, a recent meta-analysis by Moghadamnia et al., including 22 studies and 13,498 SOT recipients, found that PTLD incidence was significantly lower among those who received antiviral prophylaxis (RR 0.77; 95% CI, 0.63–0.94) [20]. In heart and kidney transplant recipients, Albatati et al. found that valganciclovir delayed viremia onset compared to no antiviral use (143 vs. 90 days; p = 0.008), with each additional day of prophylaxis increasing viremia-free survival by 1.4% (p < 0.001) [15]. In a case-control study of 100 PTLD cases and 375 controls, ganciclovir use was associated with a 38% reduction in early PTLD risk for each 30-day period during the first posttransplant year [17]. Lytic replication during primary EBV infection promotes dissemination via infected B cells and remains susceptible to antiviral suppression [31]. However, these findings do not necessarily support the use of nucleoside analogs in EBV D+/R- recipients, who are at the highest risk of primary infection. Antiviral prophylaxis is not currently recommended by current guidelines, as evidence remains limited [1]. In the absence of interventional studies evaluating preventive strategies, further research is warranted to identify approaches that reduce PTLD risk.

In our study, 60% of PTLD cases occurred in male transplant recipients, consistent with prior research indicating the disproportionate impact of PTLD, particularly late-onset disease, on males [30]. This disparity may stem from differential immune responses and increased susceptibility to EBV-associated complications [14, 32]. We also observed that patients with PTLD were older than those without, likely reflecting increased risk due to immunosenescence [33]. Most PTLD cases in our cohort were late-onset (median time from transplant to diagnosis: 6.1 years), consistent with prior studies linking late-onset PTLD to older age and early-onset PTLD to younger recipients [30].

We found an inconsistent association between PTLD and rabbit antithymocyte globulin (rATG) use, despite previous studies showing a possible link [34, 35]. This inconsistency may be partly explained by selective survival bias: recipients at a higher risk for PTLD are less likely to receive rATG, as clinicians frequently avoid its use in these patients due to its known association with PTLD [36, 37]. Additionally, 88% of patients with PTLD in this cohort had a late-onset disease, typically many months or even years after rATG exposure.

The application of newer ML models underscores the evolving methodology in estimating PTLD risk. RFC particularly aggregates predictions from multiple decision trees to mitigate overfitting and enhance generalizability [38, 39]. These strengths make RFC particularly suitable for analyzing highly imbalanced datasets and predicting rare events [40]. Previous studies have demonstrated that RFC often outperforms LR in terms of predictive accuracy and interpretability in various medical domains, including organ transplantation [41, 42]. In our study, RFC and LR models produced comparable AUC values of approximately 0.65 in the training set and 0.61 in the testing set. Model performance was likely impacted by extreme class imbalance with PTLD occurring in only 1.5% of patients. To address this, we employed random undersampling repeating the process 20 times to balance the dataset and improve model sensitivity to minority-class predictions [21]. Although random undersampling risks excluding valuable majority-class data, this iterative approach helped achieve a balance between data retention and model focus [43]. We could not include granular clinical, dynamic, or omics data—such as medication dosages, viral load, or immune markers—which likely limited model performance. These individual-level variables are unavailable in population-based sources like the SRTR. The modest overall performance may also reflect the limited granularity of the feature set, which may not adequately capture individual-level variation in PTLD risk. Incorporating personalized variables, such as pharmacogenetic or molecular data, could improve future model performance.

Our study had some limitations. First, the retrospective cohort design is subject to inherent constraints, including the potential for selection bias. Due to considerable missing data on EBV and CMV serostatus among thoracic transplant recipients, we excluded this subgroup from the cohort. Another limitation is the small number of PTLD cases, which reduced model performance; as such, our analysis is intended to generate hypotheses and highlight potential risk patterns rather than serve as a clinical prediction tool. The predictive performance of both logistic regression and random forest models was modest (test AUC ∼0.61), which likely reflects several methodological challenges: the low incidence of PTLD, the resulting class imbalance despite undersampling, and the absence of granular clinical variables such as viral load, dynamic immune monitoring, heterogeneity in immunosuppressive regimens, donor-recipient characteristics, and center-specific protocols in the SRTR database. These limitations may have reduced the ability of our models to achieve higher discrimination.

This study demonstrates the utility of ML models in predicting the rare post-transplant outcomes such as PTLD in the SOT population. Our study may challenge traditional reliance on linear models. By bridging established research with cutting-edge methodologies, this study paves the way for improved clinical decision-making and personalized patient care. Our findings have potential implications in the PTLD risk assessment and suggest using compound [EBV (D/R), CMV (D/R)] serostatus rather than simple EBV (D/R) for PTLD prediction.

## Data Availability

Publicly available datasets were analyzed in this study. This data can be found here: The data will be available upon request and approval.

## Footnotes

1
https://www.srtr.org/about-the-data/the-srtr-database/
