# Supplementary material for: Machine Learning–Based Evaluation of Combined EBV and CMV Serostatus as Predictors of Post-Transplant Lymphoproliferative Disorder
Source: Transpl Int. 2026 Feb 11;39:15781. doi: 10.3389/ti.2026.15781 (PMC12933947; doi:10.3389/ti.2026.15781)
Supplement: Supplementary file 1 [file Supplementaryfile1.docx]

# Appendix: Machine Learning–Based Evaluation of Combined EBV and CMV Serostatus as Predictors of Post-Transplant Lymphoproliferative Disorder

Ghazal Azarfar, Muath A.M. Alotaibi, Yingji Sun , Shahid Husain, Aman Sidhu, Mamatha Bhat, Seyed M. Hosseini-Moghaddam

Supplemental eTable 1-Disease characteristics in SOT recipients with PTLD

| Variable | Patients with PTLD, n=716 (%) |
| --- | --- |
| Onset | |
| Early (< 2 years) | 143 (20.0) |
| Late (> 2 years) | 573 (80.0) |
| Extra nodal involvement, n=174 | |
| Allograft | 11 (1.5) |
| Bone marrow | 28 (3.9) |
| CNS* | 18 (2.5) |
| Colon | 24 (3.4) |
| Liver | 28 (3.9) |
| Lung | 26 (3.6) |
| Small intestine | 22 (3.1) |
| Stomach | 17 (2.4) |
| Pathology type | |
| Polymorphic Hyperplasia | 6 (0.8) |
| Polymorphic PTLD (lymphoma) | 108 (15.1) |
| Monomorphic PTLD (lymphoma) | 279 (39.0) |
| Multiple Myeloma, Plasmacytoma | 37 (5.2) |
| Hodgkin's Disease | 10 (1.4) |
| Other, Specify | 276 (38.5) |

*CNS: Central nervous system

Supplemental eTable 2- Logistic regression analysis to predict the PTLD risk

| Number | Variable | Adjusted odds ratio (95% CI) |
| --- | --- | --- |
| 1 | Age | 1.241 (1.240-1.242) |
| 2 | Male | 1.038 (1.037-1.038) |
| 3 | Female | 0.964 (0.963-0.964) |
| 4 | White | 1.330 (1.329-1.330) |
| 5 | Non-white | 1.128 (1.127-1.128) |
| 6 | Kidney | 1.000 (1.000-1.000) |
| 7 | Liver | 1.000 (1.000-1.000) |
| 8 | Kidney & Pancreas | 1.000 (1.000-1.000) |
| 9 | Cyclosporine or Tacrolimus | 1.028 (1.026-1.029) |
| 10 | mTOR inhibitor | 0.927 (0.926-0.927) |
| 11 | Corticosteroid | 0.985 (0.984-0.985) |
| 12 | Mycophenolate or Imuran/Azathioprine | 1.549 (1.546-1.551) |
| 13 | Antilymphocyte | 0.863 (0.862-0.864) |
| 14 | EBV(D-/R-), CMV(D-/R-) | 0.993 (0.992-0.993) |
| 15 | EBV(D-/R-), CMV(D+/R-) | 1.084 (1.082-1.085) |
| 16 | EBV(D-/R-), CMV(D-/R+) | 0.978 (0.976-0.979) |
| 17 | EBV(D-/R-), CMV(D+/R+) | 1.013 (1.012-1.014) |
| 18 | EBV(D+/R-), CMV(D-/R-) | 1.238 (1.237-1.238) |
| 19 | EBV(D+/R-), CMV(D+/R-) | 1.142 (1.141-1.142) |
| 20 | EBV(D+/R-), CMV(D-/R+) | 1.102 (1.101-1.102) |
| 21 | EBV(D+/R-), CMV(D+/R+) | 1.021 (1.020-1.021) |
| 22 | EBV(D-/R+), CMV(D-/R+) | 1.030 (1.029-1.030) |
| 23 | EBV(D-/R+), CMV(D+/R-) | 0.669 (0.668- 0.669) |
| 24 | EBV(D-/R+), CMV(D-/R+) | 1.002 (1.001-1.002) |
| 25 | EBV(D-/R+), CMV(D+/R+) | 1.010 (1.009-1.010) |
| 26 | EBV(D+/R+), CMV(D-/R-) | 1.004 (1.003-1.004) |
| 27 | EBV(D+/R+), CMV(D+/R-) | 0.900 (0.899-0.900) |
| 28 | EBV(D+/R+), CMV(D-/R+) | 0.969 (0.968-0.969) |
| 29 | EBV(D+/R+), CMV(D+/R+) | 1.014 (1.013-1.014) |

Supplemental eTable 3- List of variables included in multivariate analysis for PTLD prediction

| Variable | Type | Categories |
| --- | --- | --- |
| Age | Numeric |  |
| Sex | Categorial | Male, Female |
| Race | Categorial | White, Non-white |
| Transplant organ | Categorial | Kidney, Liver, Kidney & Pancreas |
| Recent Immunosuppressant | Categorial | Cyclosporine Vs Tacrolimus, Mycophenolate Vs Imuran/Azathioprine,  mTOR inhibitor,  Antilymphocyte globulin,  Corticosteroid |
| EBV-CMV serology | Categorial | EBV(D-/R-), CMV(D-/R-)  EBV(D-/R-), CMV(D+/R-)  EBV(D-/R-), CMV(D-/R+)  EBV(D-/R-), CMV(D+/R+)  EBV(D+/R-), CMV(D-/R-)  EBV(D+/R-), CMV(D+/R-)  EBV(D+/R-), CMV(D-/R+)  EBV(D+/R-), CMV(D+/R+)  EBV(D-/R+), CMV(D-/R+)  EBV(D-/R+), CMV(D+/R-)  EBV(D-/R+), CMV(D-/R+)  EBV(D-/R+), CMV(D+/R+)  EBV(D+/R+), CMV(D-/R-)  EBV(D+/R+), CMV(D+/R-)  EBV(D+/R+), CMV(D-/R+)  EBV(D+/R+), CMV(D+/R+) |


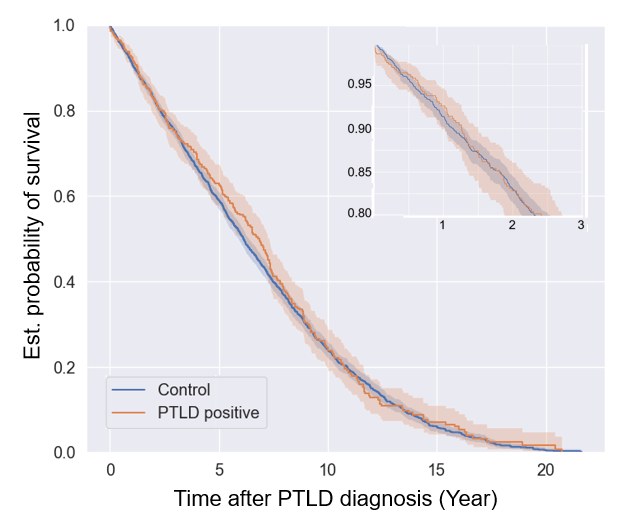


Supplemental Figure 1. Kaplan-Meier estimator for survival. Overall survival of patients with PTLD after transplant compared with patients without PTLD. Patient are matched using propensity score matching for transplant year, age, sex, race, organ transplanted, immunosuppression and EBV/CMV serostatus.
